# The unresolved struggle of 16S rRNA amplicon sequencing: a benchmarking analysis of clustering and denoising methods

**DOI:** 10.1186/s40793-025-00705-6

**Published:** 2025-05-13

**Authors:** Mohamed Fares, Engy K. Tharwat, Ilse Cleenwerck, Pieter Monsieurs, Rob Van Houdt, Peter Vandamme, Mohamed El-Hadidi, Mohamed Mysara

**Affiliations:** 1 Bioinformatics Group, Center for Informatics Science, School of Information Technology and Computer Science, Nile University, Giza, Egypt; 2 Veterinary Research Institute, National Research Centre, Giza, Egypt; 3 Laboratory of Microbiology, Department of Biochemistry and Microbiology, Faculty of Sciences, BCCM/LMG Bacteria Collection, Ghent University, Ghent, Belgium; 4 Department of Biomedical Sciences, Institute of Tropical Medicine Antwerp, Antwerp, Belgium; 5 Microbiology Unit, Nuclear Medical Applications, Belgian Nuclear Research Centre (SCK CEN), Mol, Belgium; 6 Laboratory for Microbiology, Department of Biochemistry and Microbiology, Ghent University, Ghent, Belgium

**Keywords:** Operational taxonomical units (OTUs), Amplicon sequence variants (ASVs), 16S rRNA amplicon sequencing, Denoising.

## Abstract

**Background:**

Although 16S rRNA gene amplicon sequencing has become an indispensable method for microbiome studies, this analysis is not error-free, and remains prone to several biases and errors. Numerous algorithms have been developed to eliminate these errors and consolidate the output into distance-based Operational Taxonomic Units (OTUs) or denoising-based Amplicon Sequence Variants (ASVs). An objective comparison between them has been obscured by various experimental setups and parameters. In the present study, we conducted a comprehensive benchmarking analysis of the error rates, microbial composition, over-merging/over-splitting of reference sequences, and diversity analyses using the most complex mock community, comprising 227 bacterial strains and the Mockrobiota database. Using unified preprocessing steps, we were able to compare DADA2, Deblur, MED, UNOISE3, UPARSE, DGC (Distance-based Greedy Clustering), AN (Average Neighborhood), and Opticlust objectively.

**Results:**

ASV algorithms—led by DADA2— resulted in having a consistent output, yet suffered from over-splitting, while OTU algorithms—led by UPARSE—achieved clusters with lower errors, yet with more over-merging. Notably, UPARSE and DADA2 showed the closest resemblance to the intended microbial community, especially when considering measures for alpha and beta diversity.

**Conclusion:**

Our unbiased comparative evaluation examined the performance of eight algorithms dedicated to the analysis of 16S rRNA amplicon sequences with a wide range of mock datasets. Our analysis shed light on the pros and cons of each algorithm and the accuracy of the produced OTUs or ASVs. The utilization of the most complex mock community and the benchmarking comparison presented here offer a framework for the comparison between OTU/ASV algorithms and an objective method for the assessment of new tools and algorithms.

**Supplementary Information:**

The online version contains supplementary material available at 10.1186/s40793-025-00705-6.

## Background

16S rRNA amplicon sequencing is a powerful tool to infer the microbial composition of a given sample [1, 2]. This genomic marker is sequenced with appropriate depth to identify the microbial members and their relative abundance. However, this approach is vulnerable to biases and technical errors introduced during several steps of this protocol, such as contaminant sequences, PCR point errors, chimeric artificial sequences, and sequencing errors [3–6]. Sequencing errors, in particular, are platform-dependent, with Illumina sequencing primarily exhibiting nucleotide substitutions rather than indel errors seen with other platforms. These substitutions often stem from high correlation in signal intensities, which are also influenced by signal dependency between cycles or the presence of the GC-rich motifs [4]. Consequently, the presence of erroneous reads and artifacts affects the observed diversity drastically, thereby posing a significant challenge to the identification of biological reads that truly represent the members of the microbial community members [7, 8].

Typically, clustering reads based on their identity (usually 97%) into a single taxon has been used to overcome this sequencing noise, which is commonly referred to as an operational taxonomical unit (OTU). This approach assumes that these variants originate from one genuine biological sequence that has been affected by errors introduced during the sequencing process [9, 10]. Among the clustering-based methods, UPARSE and VSEARCH-DGC implement a greedy clustering algorithm to construct the OTU structure [11, 12]. On the other hand, mothur software calculates a distance matrix that is clustered with a fixed similarity cutoff using either nearest, furthest, or average neighbor methods [10]. A recent algorithm in mothur, named Opticlust, assembles the clusters iteratively, evaluates their quality through the Matthews correlation coefficient, and consequently merges, relocates, or assigns as a novel cluster [13].

Despite the advancements in those clustering algorithms, concerns remain about the limitations of utilizing a rigid clustering cutoff [14]. Previous work demonstrated that the clustering cutoff should be region-specific and taxa-dependent, as applying a more stringent cutoff might encompass distinct taxonomical variations, while a more relaxed cutoff could fail to capture meaningful biological insights [15, 16]. Nonetheless, applying such dynamic cutoffs is dependent on the extent to which a microbial niche is studied, leaving the 3% cutoff as the default for most clustering algorithms [17–25].

More recent methods have emerged to address this problem by producing Amplicon Sequence Variants (ASVs). These techniques rely on different statistical models to discriminate real sequences from spurious ones. Tools such as DADA2, MED, UNOISE3, and Deblur adopt this approach. DADA2 implements an iterative process of error estimation and partitioning sequences based on the model implemented [26, 27]. Minimum Entropy Decomposition (MED) relies on a similar iterative premise by detecting sequence-position entropies not likely to be explained by errors [28, 29]. Deblur employs a pre-calculated statistical error profile to estimate the position’s likelihood of being erroneous and corrects it accordingly [30]. UNOISE3 compares the abundance of reads to similar sequences and then collapses identical reads into error-free and erroneous categories, utilizing a probabilistic model to assess insertion and substitution probabilities for denoising. Such denoising approaches are presumed to improve the taxonomical resolution by differentiating biological differences on a single-nucleotide level. An apparent advantage is the consistency of ASVs as sequence labels that can be used across studies without the need for re-clustering, in contrast with OTU approaches [16]. Nevertheless, this method introduces other problems, for instance generating several ASVs for non-identical 16S rRNA gene copies within the same strain. More recent methods emerged to address this problem by producing Amplicon Sequence Variants (ASVs), Exact Sequence Variants (ESVs), or zero radius OTUs (zOTUs), which are all referring to the same concept. These techniques rely on different statistical models to discriminate real sequences from spurious ones. Further details on the various OTUs/ASVs approaches are provided in Additional File 1.

Several studies have compared their developed clustering and denoising pipelines to existing alternatives; however, few independent studies have comprehensively compared clustering methods without focusing solely on algorithmic concepts or including comparisons at the ASV algorithm level [31–38]. Consequently, varying filtering criteria and chimera removal methods within these pipelines obscured a clear understanding of the performance of the various clustering/denoising approaches [33]. Another challenge comes with the selection of the appropriate dataset for the comparative analysis, as argued by Bokulich et al. [39]. Although numerous microbial community samples exist, the absence of the ground truth impedes our ability to properly evaluate the clustering/denoising approaches [27, 35, 40, 41]. While mock community data is an appropriate alternative, available mock datasets do not capture the complexity naturally found in diverse biological environments [see Additional file 2]. Thus, there is a need for an objective comparative analysis utilizing a more complex mock sample able to independently evaluate the strengths and limitations of the clustering/denoising approaches.

In this work, we aim to subject clustering and denoising approaches to an unbiased and challenging head-to-head comparison and to highlight the strengths and limitations of each approach [see Additional file 1]. In particular, we will explore their differences by predicting the composition of the different mock communities, their error rate, merging/splitting behavior, diversity analyses, and run-time analysis. For this purpose, we used 16S rRNA amplicon sequencing data generated from the most complex microbial mock community to date, consisting of 227 bacterial strains from 197 different species [42] covering V3-V4 region. In addition, we included a plethora of publicly available mocks that cover V4 region alone to enrich our comparative analysis and to further illustrate the differences between the above-mentioned algorithms.

## Methods

### Mock data

Two main data sources were used in this study. The first dataset, HC227_V3V4, was generated by amplifying the HC227 mock community with primers targeting the V3–V4 variable region of the 16S rRNA gene (5’-CCTACGGGNGGCWGCAG-3’ and 5’-GACTACHVGGGTATCTAATC-3’ as forward-reverse primer pair). Sequences were obtained on an Illumina MiSeq4000 platform in a 2 × 300 bp paired-end run (Illumina Inc., San Diego, CA, USA). The HC227 mock community consists of genomic DNA from 227 bacterial strains belonging to 197 different species [42]. The second dataset consisted of thirteen   16S rRNA gene amplicon datasets [see Additional file 3] collected from the Mockrobiota database [39]. Paired-end Illumina-MiSeq mock data sets were collected along with their expected reference sequences and taxonomic reference composition. The mocks covered a wide spectrum of input diversity ranging from 15 to 59 bacterial species; we selected the mock samples covering the V4 region of the 16S rRNA gene to reduce discrepancies. The 16S rRNA gene-targeted regions of the reference species within HC227_V3V4 and Mockrobiota [39] mock communities were dereplicated using the *unique.seqs* command in mothur (v.1.43.0) [10], which yielded distinct reference-variants referred to as *ASV-ref.* Subsequently, these unique sequences for both datasets were clustered into OTUs, which we referred to as *OTU-ref*.

### Data preprocessing

Sequence quality was checked using FastQC (v.0.11.9, Babraham Bioinformatics); primer sequences were stripped using the cutPrimers (v 2.0) tool [43]. Paired-end reads were merged using the *fastq_mergepairs* command in USEARCH (v 11.0.667) [44], and length trimming was achieved using PRINSEQ tool (v 0.2.4) and FIGARO [45, 46]. Misoriented reads were aligned to the SILVA database (Release 132) [47, 48], and incorrectly oriented (i.e., flipped) reads were filtered out using the *screen.seqs* command in mothur (v.1.43.0) [10]. Further quality filtration was performed using the USEARCH (v11.0667) *fastq_filter* command to discard all reads possessing ambiguous characters as well as optimize the maximum error rate *fastq_maxee_rate* = 0.01 [44]. Unlike the rest of the tools, DADA2 read merging occurs towards the end of the filtering steps. Thus, to detangle the effect of merging from the denoising/clustering effect (our main objective), reads were analyzed in two scenarios, one using only the forward reads as single-end (SE) reads and a second with merged forward and reverse paired-end reads (PE). This made it possible to assess whether the observed performance is different between PE and SE reads. Mock samples were subsampled to 30,000 reads per sample using the mothur *sub.sample* command to have a reasonable level of errors/artifacts.

### Methods included in the comparison

We compared the performance of four ASV denoising approaches, i.e., DADA2 (v 1.16) [27], Deblur (v 1.1.0) [30], MED (v 2.0) [29], and UNOISE3 (v11.0.667) [49], and four clustering methods, i.e., UPARSE (v11.0.667) [11], average neighborhood (AN) (v 1.43.0) [10], Opticlust (v 1.43.0) [13], and VSEARCH (v 1.43.0) [12]. All the parameters set for each algorithm can be found in Additional file 4.

### Sequence error correction and OTU generation

For DADA2, the error model was trained using the *learnErrors* command, and sequence variant inference was done using the core command *dada* (setting *OMEGA_C* parameter was set to 0). As DADA2 is highly dependent on its own preprocessing, another set of the results is done using the default parameters together with the unified parameters. In Deblur, filtering was performed using the default Deblur positive mode, and biom tables were built using chimera retained denoised files. For MED, the default pipeline command *decompose* was applied with the minimum substantive abundance parameter set to one, *relocate-outliers* and *skip-removing-outliers* options were turned on to disable the removal of outlier and low abundance reads.

For UPARSE and UNOISE3, the *fastx_uniques* command was used to identify unique sequences along with their abundances using default parameter settings. In UPARSE, the reads were clustered using the *cluster_otus* command. In UNOISE3, reads were denoised using the *unoise3* command. For both, the *minsize* parameter was set to one, chimeric reads were retained, and the OTU table was generated using the *otutab* command.

### Clustering workflow for mothur algorithms

The Average neighborhood, VSEARCH (calculated using distance-based greedy clustering; DGC), and Opticlust methods were used within the mothur pipeline. For all three methods, input FASTA formatted files were dereplicated using the *unique.seqs* command and unique read counts were reported using the *count.seqs* command. Preliminary clustering was performed using the *pre.cluster* command and the pairwise distance matrix was calculated using the *dist.seqs* command. The three clustering approaches were applied separately using the *cluster* command with a 0.03 distance cutoff.

**Fig. 1:**
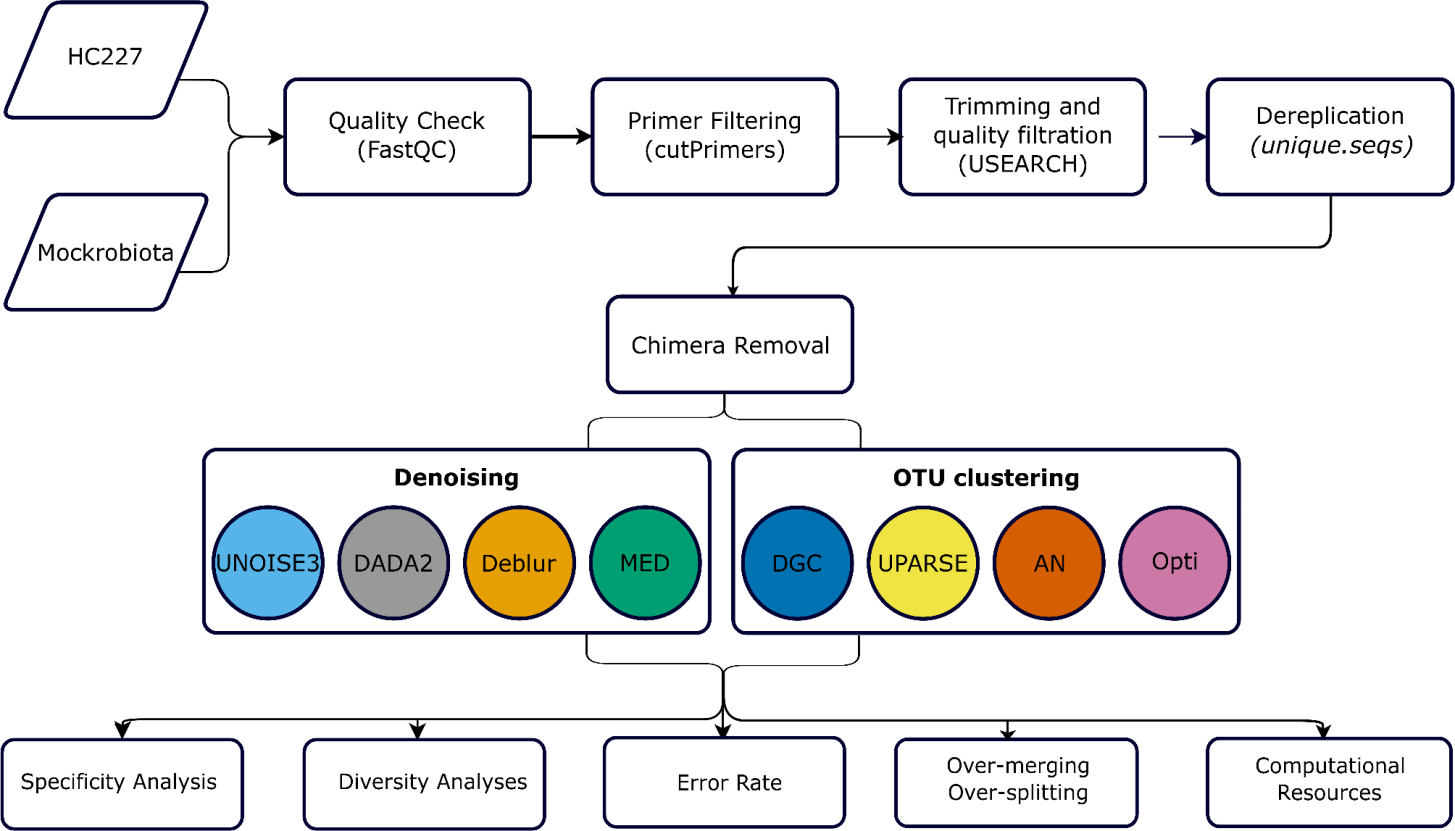
Overall workflow of benchmarking steps. The process starts from data pre-processing, followed by dereplication, denoising or clustering and chimera removal, ending with OTUs & ASVs tables and evaluation comparisons performed on them

Finally, shared files were generated using *make.shared* for each method and OTU FASTA files were generated using the *get.oturep* with the method parameter set to *abundance*. To standardize the comparison, chimera removal method was unified using the *seq.error* command against the mock’s reference sequences to avoid discrepancies between chimera-removal algorithms. Additionally, singleton reads, i.e., reads with only one occurrence, were retained for all methods (Fig. 1).

### Analysis and output comparison

We applied several criteria to evaluate the results obtained from the different algorithms. Considering that each approach received the same input (after the unified pre-processing), the generated OTUs/ASVs could be put head-to-head against several criteria, namely microbial composition, error rate, and OTUs/ASVs boundary definition. Also, Shannon and Observed Feature indexing for alpha diversity were calculated using the *summary.single* command in mothur. Beta diversity was calculated using commands *dist.shared* and *pcoa*, respectively. To further assess sequencing depth, rarefaction curves were generated, and alluvial plots were created to visually represent the diversity outputs of each algorithm compared to both ASV-ref and OTU-ref ground truth versions.

### Specificity analysis

OTU tables generated by different approaches were parsed into the mothur count file format and representative OTU sequences were generated. Subsequently, these OTUs/ASVs were categorized into four groups according to reference sequence identity: exact match (100% sequence identity with the reference sequence), mismatch (> 97% sequence identity with the reference sequence), contamination (< 97% sequence identity with the reference sequence and > 97% sequence identity with a SILVA database entry), and others (all not classified above). The sequence identity was calculated using standalone BLAST [v 2.10.0; 50] with query coverage set to 100%.

### Error rate calculation

The PCR amplification and sequencing process inherently poses the possibility of introducing several biases, such as PCR single-base mistakes, PCR chimeras, and sequencing errors. According to recent work [51], it is essential to carefully identify and eliminate these mistakes as part of the analytical process. The research results may be skewed if these biases are not adequately addressed, which could result in a large, inaccurate estimation of the diversity of the microbial community. For each algorithm, chimeric reads were determined using the mothur command *seq.error* command and removed before the error rate of the OTUs/ASVs was calculated for each mock. The error rates of the OTUs/ASVs were assessed against reference sequences in terms of mismatches, insertions, and deletions and calculated by comparing the number of mismatched positions to the total number of bases for each mock.

### Merging/splitting analysis

Furthermore, the over-merging rates (where more than one reference sequence was merged into the same OTU/ASV) and over-splitting frequencies (where the same reference sequence was split into multiple OTUs/ASVs) were evaluated. For this purpose, the OTUs/ASVs were mapped against the unique and clustered reference sequences (i.e., *ASV-ref* and *OTU-ref,* respectively) using standalone BLASTn (v 2.10.0) [50], setting query coverage to 100% and percent identity to 97%. Reference sequences that were found absent, albeit being present prior to the denoising/clustering step, were considered over-merged. Moreover, if the same reference sequence was mapped to more than a single OTU/ASV, it was considered over-split. Furthermore, a head-to-head comparison was done comparing the abundance of each bacterial strain in the HC227_V3V4 mock community against the number of sequence reads aligned to it using compatible ASV/OTU reference versions.

### Diversity analysis

To compare the diversity of observed OTUs/ASVs across different approaches, we generated rarefaction curves using the *rarefaction.single* command in mothur. In terms of diversity analysis, we calculated alpha diversity using Shannon indexing [52], which considers both the abundance and evenness of species present and Observed Feature (Sobs Calculator) indexing [52], which only considers evenness. The *summary.single* command in mothur was utilized for calculating both indexing metrics. For beta diversity, we calculated distances between the various approaches and the theoretical *(*i.e., expected) *ASV-ref* and *OTU-ref* abundance using mothur *summary.single*, and *dist.shared* commands then we compared generated coordinates by PCoA plotting using *pcoa* command. We accommodated Jclass and Euclidean distance metrics [53, 54] that focus on the presence or absence of ASVs rather than their abundance and Canberra similarity calculation [55, 56] that consider both presence/absence and abundance. Moreover, we conducted a direct comparison of beta diversity distances between the algorithms against both our reference versions (i.e., OTU-ref and ASV-ref) as benchmarks.

### Computational cost analysis and clustering parameter effect

To evaluate the runtime and memory consumption of each clustering/denoising algorithm, we conducted tests using subsampled, preprocessed reads from the HC227_V3V4 mock community. Using different sets of 5000 to 20,000 reads per sample utilizing the *sub.sample* command in mothur with varying size parameters. Each algorithm was executed on a PC equipped with 64 GB of RAM and 12 threads, with multithreading enabled across all algorithms. Moreover, we tested cutoffs ranging between 0.01 and 0.05 for clustering-based approaches, with exception of UPARSE, as it was not possible to change the hardcoded cutoff.

### Statistical analysis

We statistically evaluated the error rate, specificity, and merging/splitting differences across various OTU/ASV algorithms. Normality of the data was assessed using the Shapiro-Wilk test. For normally distributed data, we applied ANOVA followed by Tukey’s HSD test, whereas for non-normally distributed data, we used the Kruskal-Walliss test followed by the Wilcoxon rank-sum test. A significance level of 0.05 was applied to corrected p-values. All statistical analysis and graphs were constructed using R (v.4.2.2), patchwork [57], ggplot2 [58], ggalluvial [59], and tidyr [60] packages. Codes and scripts for analysis and visualization are available at GitHub page: https://github.com/MOFares-Bioinf/BACDAS.

## Results

### DADA2 and UPARSE provide the most accurate estimate of microbial composition

**Fig. 2:**
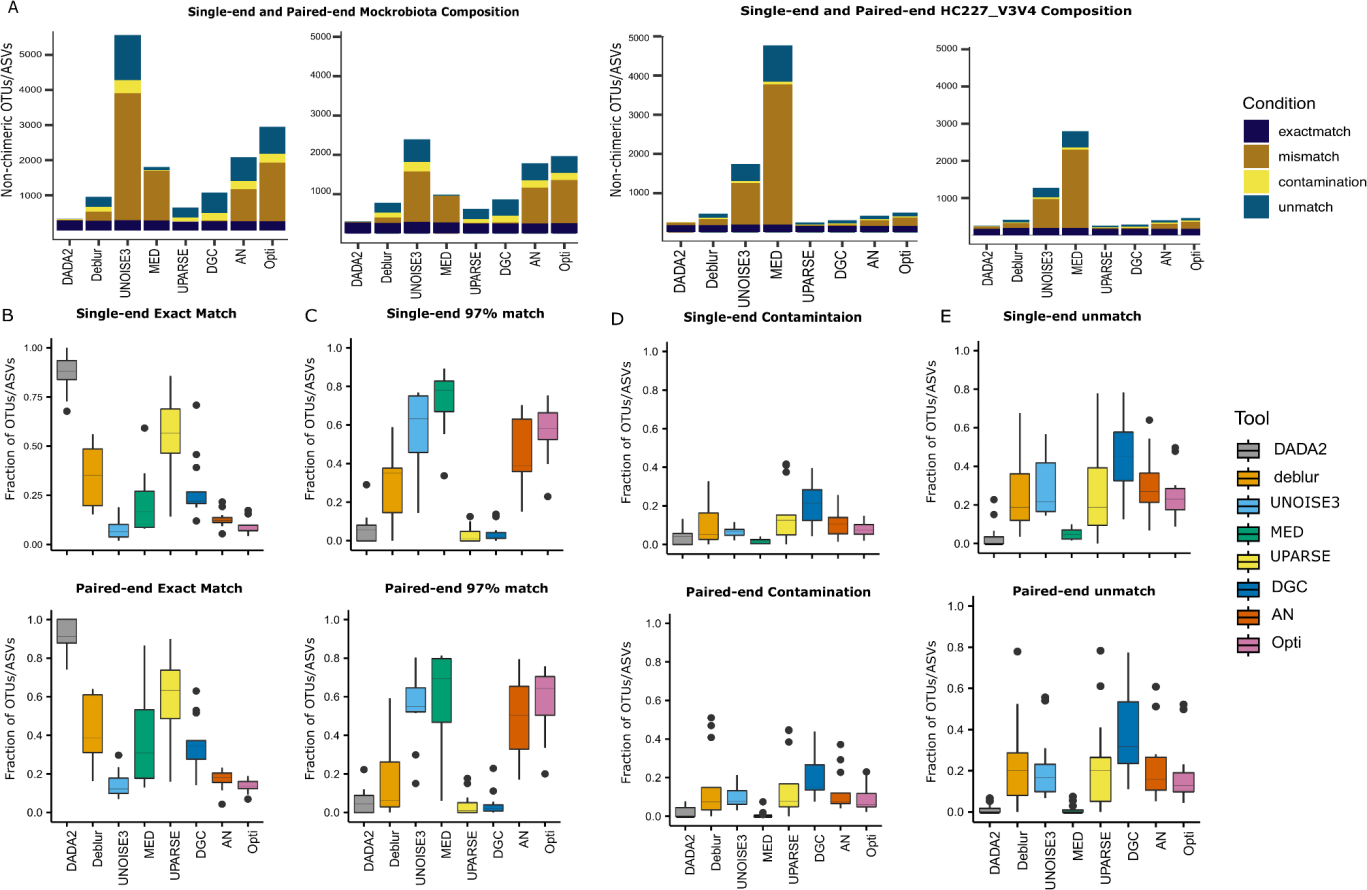
Illustrating Performance of the various algorithms in respect to the microbial composition. Stacked bar plots of OTUs/ASVs output composition for single-end and paired-end reads from Mockrobiota and HC227_V3V4, respectively **(A)**. Average box plots showing the fraction of exact matches **(B)**, 97% matches **(C)**, contaminants **(D)** and unmatched **(E)** for single-end and paired-end reads for the Mockrobiota mock community

The performance of the various denoising/clustering approaches in terms of specificity analysis was assessed using the HC227_V3V4 and Mockrobiota mocks in the SE and PE scenarios. For the HC227_V3V4 dataset, DADA2 (whether using the unified or the default parameters) and UPARSE had the highest count of exact matches, followed by DGC, AN, Opticlust, and Deblur. This was consistent for both single-end and paired-end reads and in agreement with the results from the collection of 13 mocks within the Mockrobiota community, except for DGC, AN, and Opticlust (Fig. 2A-B; Additional file 5; Supplementary Fig. 1).

DADA2 had 6% of its ASVs categorized as mismatches for the Mockrobiota community, which was higher compared to the 1% and 2% observed for UPARSE and DGC, respectively (Fig. 2C). Nonetheless, MED and UNOISE3 had the highest percentage of mismatches for the HC227_V3V4 mock community, with percentages of 75% and 61%, respectively (Fig. 2A). This outcome was consistent with the findings observed within the Mockrobiota dataset (Fig. 2C; Additional file 5). Statistical validations across the eight algorithms for each category in the Mockrobiota dataset are provided in Additional file 5. When looking into DADA2 using the default parameters, mismatch level was higher for both Mockrobiota and HC227_V3V4 mock communities relative to DADA2 unified parameters (Supplementary Fig. 1– A& C).

When examining the OTUs/ASVs identified as potential contaminants, approximately 3% were detected by DADA2 (in unified and default parameters), MED, and UNOISE3 in the Mockrobiota mock community. In contrast, UPARSE and DGC identified fewer contaminants, with less than 10%, in the Mockrobiota community compared to the HC227_V3V4 mock community. The results were consistent for both single-end and paired-end reads (Fig. 2D; Supplementary Fig. 1 - C). Finally, all approaches achieved approximately the same count of unmatched reads, around 20 ± 4%, except DADA2, that performed better with only 5% for HC227_V3V4 (Fig. 2A). Yet, this was not consistent for DGC within the Mockrobiota dataset, where it surged above 40%, and MED achieved below 5% unclassified OTUs (Fig. 2E).

### DADA2 and UPARSE showed the lowest error rate yet still with some over-merging in the ***ASV-ref***

**Fig. 3:**
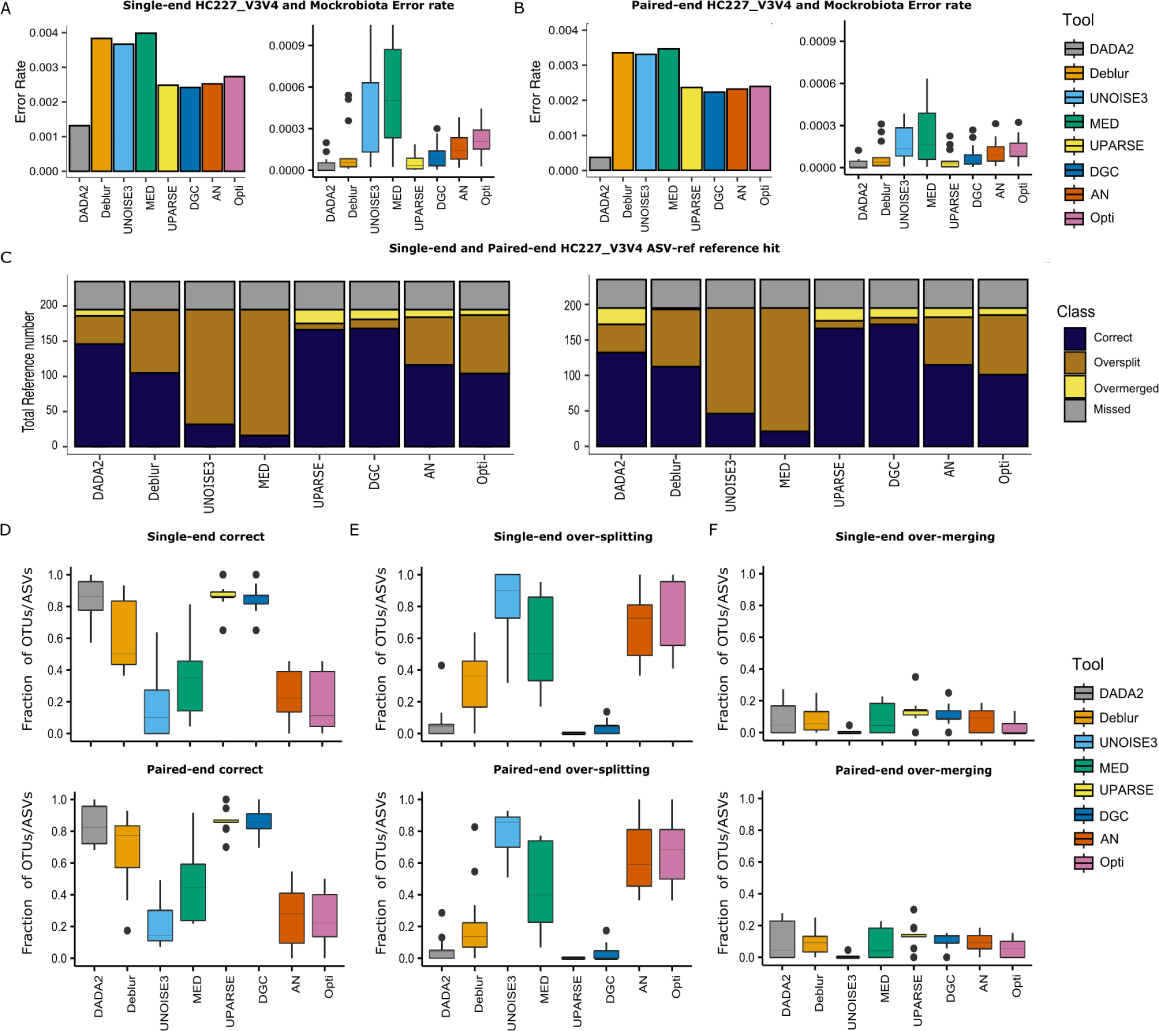
Illustration of various algorithms performance for evaluating error rate and merging/splitting based ***ASV-ref***. **A**, **B**) Bar plots demonstrate the error rate for HC227_V3V4, and Box plots demonstrate the error rate for Mockrobiota community across the algorithms for single-end and paired-end reads. **C**, ) Stacked bar plots demonstrates single-end and paired-end for total number of references in the *ASV-ref* for Mockrobiota and HC227_V3V4 respectively. **D**) Average box plots demonstrate correct percentage for single-end and paired-end Mockrobiota community, **E**) Average box plots demonstrate over-splitting percentage for single-end and paired-end reads for Mockrobiota community, **F**) Average box plots demonstrate Over-merging percentage for single-end and paired-end reads for Mockrobiota community

Next, the error rate of the OTUs/ASVs from the various clustering/denoising approaches was assessed (Fig. 3: A-B). DADA2, UPARSE, and DGC demonstrated the lowest overall value, while MED and UNOISE3 exhibited the highest error rate for both the HC227_V3V4 and Mockrobiota community datasets (Fig. 3A-B). Interestingly, Deblur’s performance was inconsistent between the two datasets, with a high error rate for the complex HC277_V3V4 mock and a much lower error rate for the Mockrobiota mocks. For DADA2, the error rate of both datasets increased when utilizing the default parameters instead of the unified parameters (Supplementary Fig. 1-B). The AN and Opticlust algorithms showed approximately the same error rate for both datasets. Statistical validation was performed for Mockrobiota dataset, ensuring the robustness of the results.

In addition, by comparing the OTUs/ASVs against the mock reference sequences, it was possible to assess whether each reference variant was mistakenly grouped with others into the same OTU/ASV (over-merged) or split over multiple OTUs/ASVs (over-split; Fig. 3: C-F). Regarding the number (and fraction) of correctly assigned OTUs/ASVs, UPARSE and DGC showed the highest performance, followed by DADA2 for both communities (Fig. 3: C, D). On the other hand, MED, UNOISE3, and Deblur identified the lowest number of correctly assigned ASVs for both datasets, while Opticlust and AN showed a slightly better performance for HC227_V3V4 and Mockrobiota Community. Similar results were reported for over-splitting, with UPARSE showing a marginal to negligible over-splitting followed by DGC and DADA2 for both datasets (Fig. 3: C, E).

Most approaches suffered from varying degrees of over-merging, except for Opticlust and UNOISE3, which showed less to approximately no over-merging in both datasets. Yet, the worst performance regarding the over-merging was achieved by MED followed by DADA2 which showed a noticeably higher level of over-merging when relying on paired-end than on single-end reads (Fig. 3: C, F). The performance of DADA2 deteriorated when utilizing default parameters, showing higher levels of over-merging for Mockrobiota mock community and higher levels of over-splitting with a slight reduction in correctly identified reads in HC227_V3V4 mock community (Supplementary Fig. 1:E). Deblur showed a slightly better performance for the Mockrobiota community dataset compared to the HC227_V3V4 dataset, which was consistent with its error rate results for the Mockrobiota community.

### **UPARSE**,** DGC**,** and DADA2 had the highest accuracy with least fraction of over-splitting and over-merging in the*OTU_ref***

**Fig. 4:**
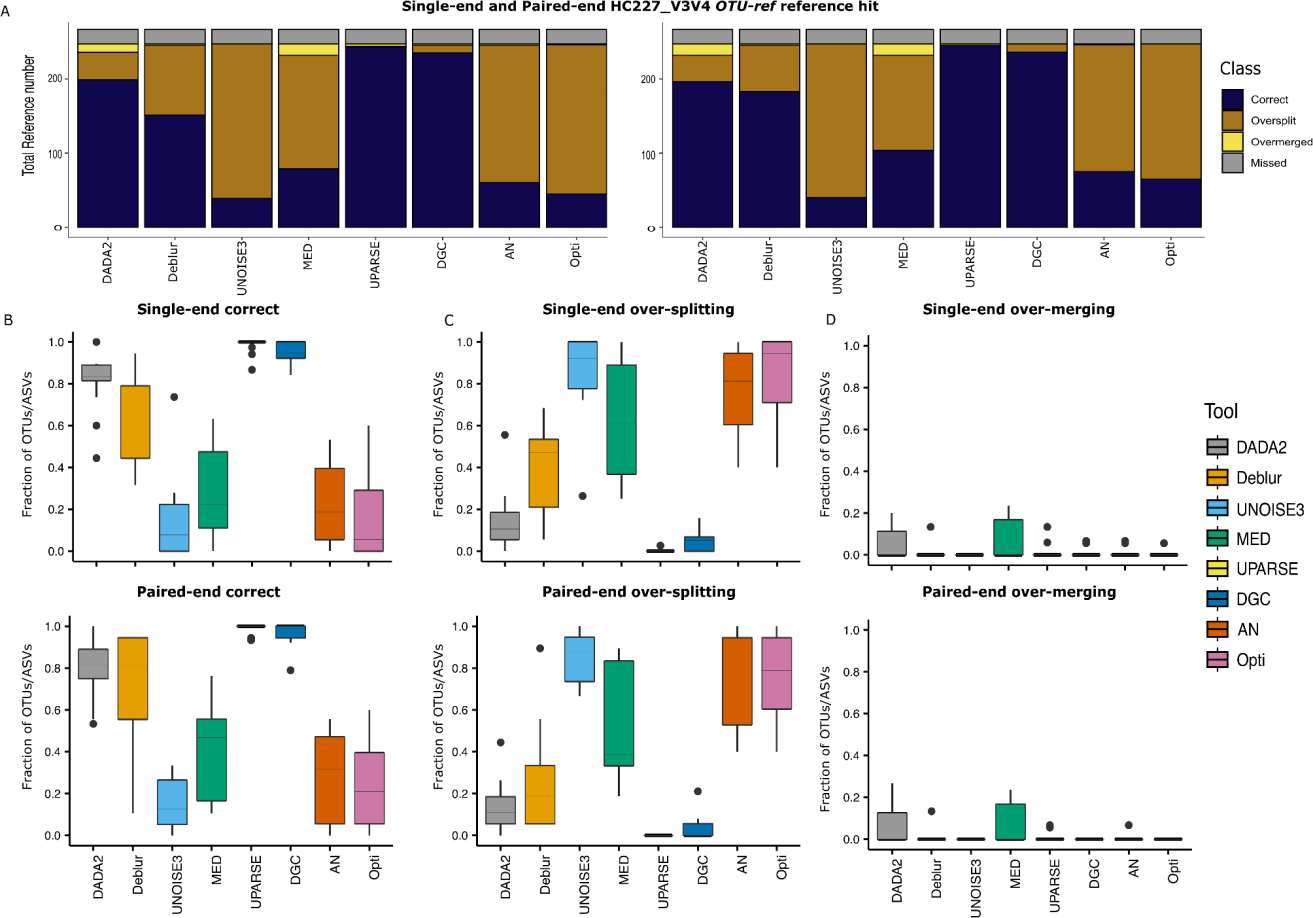
Illustration of various algorithms performance using clustering method for evaluating over-merging/over-splitting based ***OTU_ref***. **(A)** stacked bar plots represent total number of reference sequences for single-end and paired-end conditions for Mockrobiota and HC227_V3V4 datasets respectively. **B**) Average box plots representing the correct percentage for single-end and paired-end reads for Mockrobiota community **C)** Average box plots representing the over-splitting percentage for single-end and paired-end reads for Mockrobiota community, **D)** Average box plots representing the over-merging percentage for single-end and paired-end reads for Mockrobiota community

When comparing the resulting OTUs/ASVs to the clustered mock references, UPARSE, DGC, and DADA2 exhibited the highest percentage of correctly assigned reads and the lowest over-split percentage for both the Mockrobiota and HC227_V3V4 mock communities (Fig. 4 and Additional file_5 for the statistical analysis results). For AN, Opticlust, and UNOISE3 algorithms, only around 20% of the reference sequences were correctly matched with one unique OTU/ASV in the HC227_V3V4 dataset, which suggests that similar reads originating from the same reference were clustered separately (Fig. 4A). Yet, most reference sequences were split over exceeding a single OTU/ASV per reference for these algorithms.

Furthermore, Deblur showed an average performance with 35–40% of the reference sequences correctly matched with a single OTU/ASV percentage. Yet, this percentage was significantly increased for the Mockrobiota dataset (Fig. 4B). The results were mostly identical for single-end and paired-end reads [see Additional file 4]. The UNOISE3, Opticlust, and AN algorithm, with their large number of OTUs/ASVs, showed a high percentage of over-splitting reads compared to other approaches for the Mockrobiota community (Fig. 4C). Yet, UNOISE3 and MED showed the highest percentage of over-splitting OTUs/ASVs among the tested approaches for the HC227_V3V4 dataset (Fig. 4A).

Notably, 17% of HC227_V3V4 and 40% of Mockrobiota reference sequences were missed by all tested approaches. Although over-merged ASV-references were present in all clustering-based algorithms, this was effectively resolved when using *OTU-ref* as originally intended for these algorithms [see Additional file 6]. In contrast, for ASV algorithms– MED in particular- this was not the case as shown above.

After evaluating different clustering cutoffs on clustering-based algorithms compared to *OTU-ref*, cutoff 0.03 was the optimal one, yielding the highest number of correctly assigned species for all clustering approaches. Moreover, a notable increase in over-merging and decrease in over-splitting percentage was observed as lessstrict cutoff was applied [see Additional file 7].

### DADA2 and Deblur showed the closest resemblance to the mock composition

**Fig. 5:**
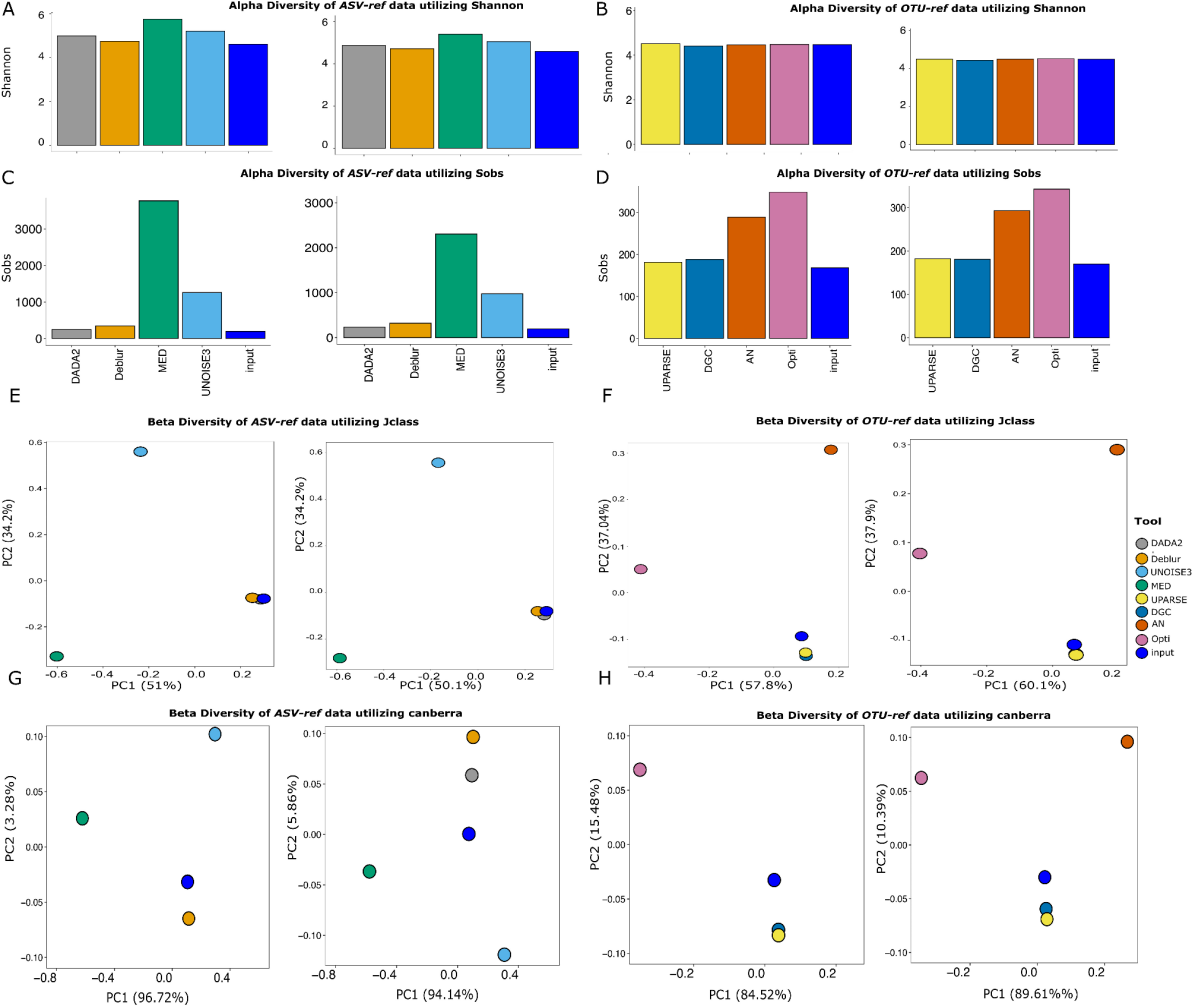
illustration of Microbial Composition, alpha and beta Diversity for HC227_V3V4 dataset. **A**, **B**) Bar plots for Shannon and Observed Feature indexing calculation for Denoising and clustering approaches compared to *ASV-ref* and *OTU-ref* theoretical input for single-end and paired-end conditions respectively. **C**, **D**) scatter plots for comparing Denoising and clustering approaches to both *ASV-ref* and *OTU-ref* theoretical input respectively using Jclass and Canberra calculation

We analyzed the composition of the HC227_V3V4 mock community and calculated the theoretical reference composition (*OTU-ref* and *ASV-ref* compatible with the OTU and ASV approaches, respectively). Regarding Shannon’s alpha diversity index, all approaches showed a very close resemblance to the *ASV-ref* or *OTU_ref*, with exception of MED and UNOISE3, which was consistent with their sub-optimal performance in respect to the reported error rates and the inflation of over-split ASVs for the same references (as shown before). For the observed feature’s richness index, DADA2 and deblur (unlike MED and UNOISE3) showed the closest microbial diversity to our input. These results were consistent with sequence mapping analysis against *ASV-ref* for the denoising algorithms [see Additional file 6]. For the clustering approaches, UPARSE showed the closest resemblance to the mock composition (*OTU-ref*), followed by DGC, AN, and Opticlust algorithms, in line with the observed feature and shannon matrices results (Fig. 5).

For beta diversity, both membership-only distances (e.g., Jclass and Euclidean matrices) and membership with abundance-based metrics (e.g., Canberra) showed that DADA2 and Deblur had the closest resemblance to the ASV-ref input (Supplementary Fig. 2), while UNOISE3 and MED exhibited less similarity and greater variance in comparison to the ASV-ref input. This was consistent for single-end and paired-end approaches (Fig. 5C). When evaluating the clustering algorithms against the OTU-ref ground truth, UPARSE exhibited the closest resemblance to the OTU-ref ground truth, with a high number of shared OTUs. This performance was followed by DGC, AN, and Opticlust. Notably, the results were consistent across both single-end and paired-end sequencing approaches (Fig. 5D).

We also compared *ASV-ref* input against denoisers and *OTU-ref* input against clustering algorithms via alluvial plots. DADA2 seems to preserve notable portion of the diversity found in the *ASV-ref* input comparedt to the other denoisers, as shown in Supplementary Fig. 3, which is consistent with the alpha diversity results (Fig.5 A).

For clustering algorithms, UPARSE seems to maintain a large amount of diversity of *OTU-ref* references and seems to balance between over-merging and over-splitting with discrepancies from the reference OTUs. Opticlust algorithm also maintains a large number of *OTU-ref*, yet is prone to over-splitting, as shown in Supplementary Figs. 2 & 3, which is also consistent with over-splitting results in Fig. 4A. The behavior of both denoising and clustering algorithms was consistent between single-end and paired-end scenarios [see Additional file 8].

### Computational resources and parameter effect exploration

Analysis of execution time, considering both CPU processing and input/output operations, showed that all algorithms performed similarly, with execution times gradually increasing as the number of input sequences grew. Yet, MED and Deblur required execution times ranging from 2 min to 120 min compared to the other algorithms, which required between 2 s and 4 min [see Additional file 9]. Furthermore, the RAM amount required for all clustering/denoising algorithms was between 5 and 11 GB of RAM, except for MED, which required up to 40 GB of RAM for a sample containing 20,000 reads. Also, a gradual increase in memory consumption was noted with the increase in the number of input sequences.

## Discussion

Defining species boundaries using 16S rRNA amplicon sequencing presents a significant challenge [15]. Analysis mainly adopts one of two approaches: either clustering similar sequences within a certain distance threshold (referred to as OTU-based approaches, such as DGC, AN, Opticlust, and UPARSE) or denoising erroneous reads (referred to as ASV-based approaches, such as DADA2, Deblur, MED, and UNOISE3). Biases and variability in different preprocessing steps make it challenging to compare such algorithms [39]. Additionally, mock community samples are typically used for this purpose, yet the available mock communities do not capture the true microbial complexity, as argued before [39]. To accommodate this, we sequenced 16S rRNA amplicons of the most complex mock community previously described in Goussarov et al. [42] and used it together with a collection of thirteen previously available mocks for a comprehensive comparative analysis. We used unified preprocessing and filtering steps to allow benchmarking the clustering/denoising algorithms objectively. As DADA2 error profiling might be sensitive to its own pre-processing, we also provided the results using the default parameters. We adopted both single-end and paired-end read scenarios in our comparison to ensure an accurate evaluation, particularly for Deblur designed for single-end reads.

Although all algorithms were allowed to identify error-free OTUs/ASVs (exactly matching the intended reference species within the mocks), they varied drastically in their ability to handle erroneous reads. DADA2, UPARSE, and DGC achieved the highest performance, with the lowest number of erroneous OTUs/ASVs and consequently the lowest number of overall OTUs/ASVs. MED and UNOISE3 had the highest number of mismatches, which exceeded the other algorithms by several folds. Interestingly, when using the default parameters, DADA2’s performance deteriorated—producing fewer ASVs that exactly matched the Mockrobiota mock community while generating additional ASVs with mismatches in both the Mockrobiota and HC227_V3V4 mock communities. This was in line with previous whole-pipeline comparative studies, where DADA2’s pipeline was more capable of identifying ASVs exactly matching the reference sequence than Deblur and UNOISE3, yet at the expense of increased over-splitting [37, 38]. Yet, in our analysis, we were able to pinpoint this phenomenon to the influence of the denoising algorithms, which was not possible previously due to the confounding effect of applying different filtering and preprocessing parameters for each approach’s pipeline. The lower percentage of contamination and unmatched sequences in denoising compared to clustering algorithms might suggest strict handling/filtering of relatively low abundance sequences, such as contamination and chimeras, by ASV approaches as also noted by Reitmeier et al., 2021 [61].

The algorithm’s efficiency in eliminating OTUs/ASVs with artifacts can also be assessed by the error rate. For instance, DADA2, UPARSE, and Deblur showed the lowest error rates in the produced OTUs/ASVs, which was consistent with their accurate assessment of the mocks’ microbial community. Yet, the error rate of DADA2 increased for both mock communities when using the default parameters compared to the unified parameters. Also, accurately correcting errors requires extensive memory usage and processing time for ASV approaches, which might be attributed to the error correction and modeling steps involved. Consequently, UNOISE3 and MED had the highest error rates, reflecting their lower performance in assessing the microbial community. Although this difference in error rate between DADA2 and MED/UNOISE3 seems minor, it had a profound effect on the biological interpretation. For instance, findings of MED and UNOISE3 diverged significantly from those produced by DADA2 and UPARSE. The former two identified the least number of correctly assigned bacterial species in addition to the largest deviation from the input data, as also reflected by both observed feature and shannon indices. The same holds for beta diversity utilizing three different distance matrices.

The other clustering-based algorithms, DGC, AN, and Opticlust performed moderately, with noticeable differences between the mean error rates of the tested mocks. Furthermore, none of the tested algorithms allowed retrieving the complete number of expected mock reference sequences, as up to 17% for HC227_V3V4 and 40% for Mockrobiota were missed by all tested approaches. This can be partly explained by the low sequence variability in the region of the 16S rRNA gene, especially after read trimming to equal length, and the presence of closely related species and strains. In addition, all tested approaches generated more OTUs/ASVs than species in the dataset, exaggerating the species richness in the sample with many false positives, even after removal of chimeric sequences. This problem, also known as OTU inflation, forms a true challenge for clustering and most denoising algorithms and has been repeatedly reported [7, 62].

UPARSE, DADA2, and DGC demonstrated the highest accuracy, each correctly assigning a single ASV/OTU per reference sequence—consistent with prior pipeline comparisons [63]. In contrast, MED and UNOISE3 performed poorly, with < 25% of reference sequences uniquely represented by a single OTU/ASV. Clustering-based algorithms frequently over-merged similar sequences (5–10% of OTUs), particularly with less stringent cutoffs [Additional file 7]. Notably, ASV-based methods like DADA2 and Deblur also exhibited over-merging (11% and 1%, respectively), likely due to erroneous sequence binning where true variants were misclassified as noise and merged into unrelated ASVs.

Validation against 97%-clustered reference sequences revealed that OTU-based algorithms recovered all expected references, whereas DADA2 missed 3% (single-end) and 10% (paired-end) of references. For paired-end data, this may reflect failed merging of heavily erroneous forward and reverse reads, while single-end losses suggest over-aggressive error filtering. DADA2 with the default parameters reduced over-merging but introduced trade-offs: increased over-splitting and fewer correctly identified ASVs. Unlike the unified parameters, which enforce strict criteria across all algorithms, the default parameters risked both discarding true biological sequences and retaining more artifacts. Collectively, ASV methods showed higher over-splitting rates than OTU approaches, indicating that over-merging and over-splitting coexist as challenges in ASV-based pipelines.

When assessing the reflection of the approaches’ varying performances on the microbial diversity, DADA2 and UPARSE (also Deblur to a lesser extent) achieved the closest resemblance -through sequence mapping- to the theoretical reference, aligning with findings from earlier studies [63–65]. This was reflected in the diversity analysis, where DADA2 and Deblur achieved the closest results to the theoretical reference. Which was observed by alpha and beta indices considering the microbial memberships solely or coupled with their abundances. However, DADA2 exhibited bacterial strain over-merging, consistent with findings of other studies [37, 66, 67]. UNOISE3 and MED on the other hand showed drastically different results from the intended theoretical diversity. For OTU-based approaches, UPARSE achieved the closest resemblance to the intended input, followed by DGC, which was manifested by both alpha and beta diversities and consistent with other pipeline comparative studies [67].

Despite the apparent differences in complexity, coverage, and the targeted 16S rRNA gene region between HC227_V3V4 and the Mockrobiota mocks, there were no discrepancies in the overall comparison conclusions. Despite our attempts to unify sequencing depth and analyze multiple depths, biases introduced by sequencing methods still pose challenges that need to be acknowledged. Furthermore, our analysis exclusively utilized Illumina data targeting the V4/V3-V4 regions of the 16S rRNA gene, yet it is still unclear whether these results will differ when using different 16S rRNA regions (V1-3, V7-9, or V4-5), long-read sequencers (e.g. PacBio) or other amplicons (e.g. ITS) [34, 68]. Thus, the robust design of our benchmark analysis, combined with the use of the complex mock community data presented, provides a strong foundation for evaluating these algorithms—a framework that could be extended to full pipeline comparisons in future studies.

## Conclusions

In conclusion, both OTU and ASV approaches produced varying results with pros and cons for each approach. ASV algorithms provided consistent sequence variants suitable for independent samples or meta-analysis studies without requiring re-clustering. DADA2 performed best among ASV approaches, preserving original diversity with little increase in over-merging, though its default parameters led to significantly higher over-splitting and mismatches. Conversely, for OTU-based algorithms, UPARSE performed the best, with balanced merging/splitting rates capable of handling inflated sequencing error/artifacts. The latter is suitable for under-examined niches or when a major microbial shift is to be expected. DADA2 and UPARSE provided the closest resemblance to the intended microbial community with the lowest error rate and the least number of artifacts.

## Electronic supplementary material

Below is the link to the electronic supplementary material.

Supplementary Material 1: fig. 1: Re-analysis of DADA2 with default parameters. DADA2 was re-analyzed to remove the confounding effect of unified preprocessing and was subsequently compared to the results of our unified preprocessing (DADA2). (A) Stacked bar plots showing specificity analysis, representing the number of non-chimeric ASVs for each condition in both the Mockrobiota and HC227_V3V4 mock communities. (B) Comparison of error rates between the unified preprocessing method (DADA2) and the default parameter method (DADA2*) for the Mockrobiota and HC227_V3V4 mock communities. (C) Box plots showing the difference in specificity analysis results between the unified preprocessing method (DADA2) and the default parameter method (DADA2*) for the Mockrobiota mock community. (D) Box plots showing the results of Merging/Splitting Analysis between the unified preprocessing method (DADA2) and the default parameter approach (DADA2*) for the Mockrobiota mock community. (E) Stacked bar plots illustrating the merging/splitting results compared to ASV-reference data for both the unified preprocessing method (DADA2) and the default parameter method (DADA2*) for the HC227_V3V4 mock community.

Supplementary Material 2: fig. 2: rarefaction curves and alluvial plots for both single-end and paired-end data. Rarefaction curves were illustrated for representing sequencing depth for denoising tools against *ASV-ref* and clustering methods against *OTU-ref* for both single-end and paired-end conditions respectively. Alluvial plots were utilized for visualizing the bacterial content across denoising and clustering algorithms for paired-end method.

Supplementary Material 3: fig. 3: illustration of HC227_V3V4 utilizing euclidian distance and alluvial plots for visualizing bacterial content for denoising and clustering methods. (A) Beta Diversity for denoising algorithms against *ASV-ref* data for both single-end and paired-end methods. (B) Beta Diversity for clustering algorithms against *OTU-ref* for both single-end and paired-end methods. C & D) stacked bar plots illustrating read count for all algorithms for Mockrobiota and HC227_V3V4 mock communities considering single-end and paired-end conditions. E) Alluvial plots were utilized for visualizing the bacterial content across denoising and clustering algorithms for single-end method.

Additional file 1: representing details about Tool characteristics comparison.

Additional file 2: represents the available mock communities with their strain number to be used in benchmarking.

Additional file 3: represents the Mockrobiota data collected from web repository of microbial mock data.

Additional file 4: Describe the parameter used for running each algorithm.

Additional file 5: Wilcoxon sum rank statistical analysis results with pairwise comparison.

Additional file 6: Theoretical and expected 16S rRNA reference HC227_V3V4.

Additional file 7: Parameter effect results on clustering-based algorithms.

Additional file 8: distance-based comparison across all clustering/denoising algorithms.

Additional file 9: Computational cost analysis results.

## Data Availability

16S rRNA amplicon sequence data generated and analyzed in this study have been deposited in the NCBI Sequence Read Archive with the accession code PRJNA975486.

## Abbreviations

OTU: Operational Taxonomic Units
ASV: Amplicon Sequence Variant
ESV: Exact Sequence Variants (ESVs)
zOTU: Zero Radius Operational Taxonomical Unit
SE: Single-End
PE: Paired-End
DADA2: Divisive Amplicon Denoising Algorithm 2
MED: Minimum entropy decomposition
AN: Average neighbor
DGC: Distance-based greedy clustering
