# Supplementary material for: The unresolved struggle of 16S rRNA amplicon sequencing: a benchmarking analysis of clustering and denoising methods
Source: Environ Microbiome. 2025 May 13;20:51. doi: 10.1186/s40793-025-00705-6 (PMC12076876; doi:10.1186/s40793-025-00705-6)
Supplement: Supplementary file 1 — Supplementary Material 1: fig. 1: Re-analysis of DADA2 with default parameters. DADA2 was re-analyzed to remove the confounding effect of unified preprocessing and was subsequently compared to the results of our unified preprocessing (DADA2). (A) Stacked bar plots showing specificity analysis, representing the number of non-chimeric ASVs for each condition in both the Mockrobiota and HC227_V3V4 mock communities. (B) Comparison of error rates between the unified preprocessing method (DADA2) and the default parameter method (DADA2*) for the Mockrobiota and HC227_V3V4 mock communities. (C) Box plots showing the difference in specificity analysis results between the unified preprocessing method (DADA2) and the default parameter method (DADA2*) for the Mockrobiota mock community. (D) Box plots showing the results of Merging/Splitting Analysis between the unified preprocessing method (DADA2) and the default parameter approach (DADA2*) for the Mockrobiota mock community. (E) Stacked bar plots illustrating the merging/splitting results compared to ASV-reference data for both the unified preprocessing method (DADA2) and the default parameter method (DADA2*) for the HC227_V3V4 mock community. [file 40793_2025_705_MOESM1_ESM.pdf]

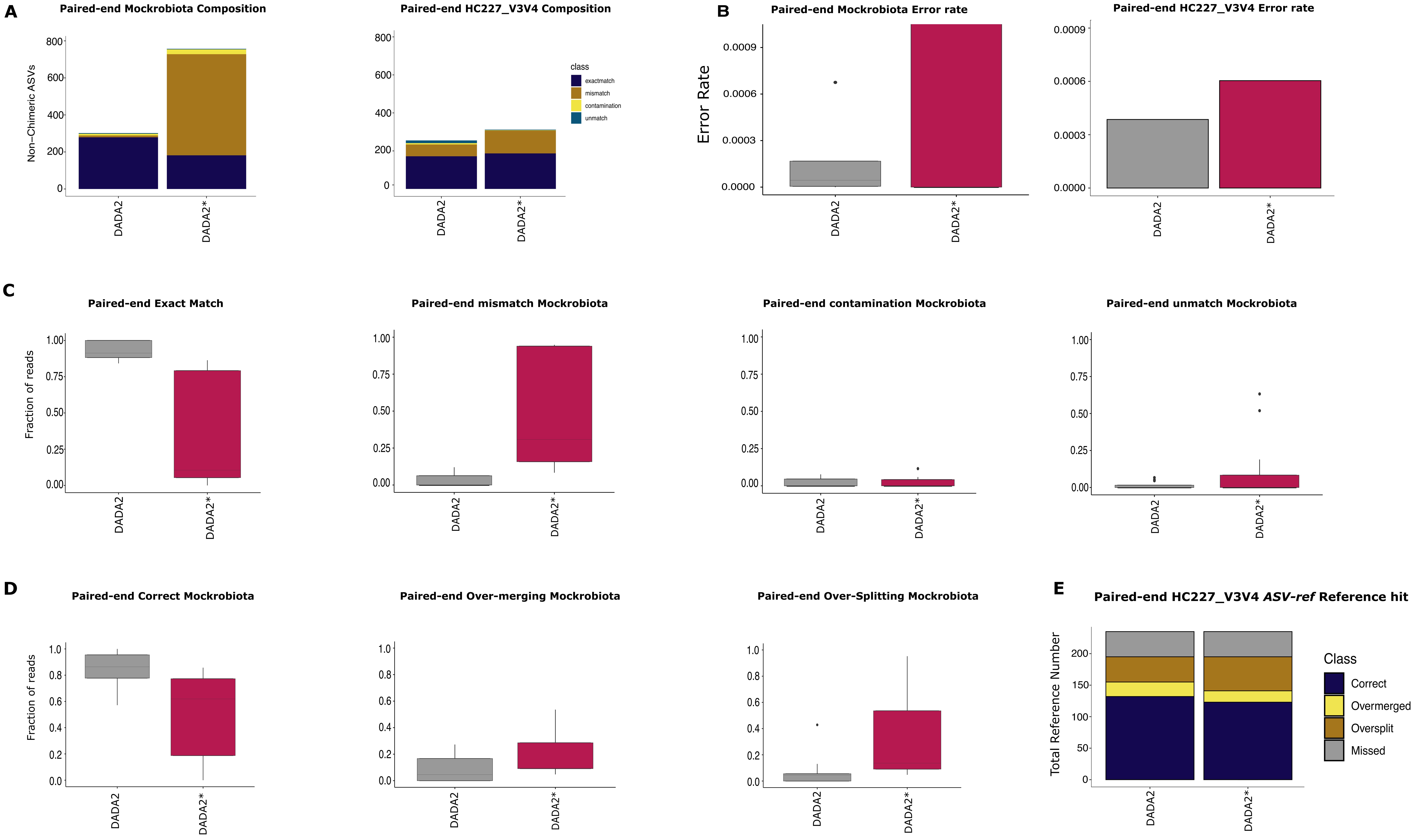
A
Paired-end Mockrobiota Composition
Non−Chimeric ASVs
0
200
400
600
800
DADA2
DADA2*
Paired-end HC227_V3V4 Composition
class
exactmatch
mismatch
contamination
unmatch
B
Paired-end Mockrobiota Error rate
Error Rate
0.0000
0.0003
0.0006
0.0009
Paired-end HC227_V3V4 Error rate
C
Paired-end Exact Match
Fraction of reads
0.00
0.25
0.50
0.75
1.00
Paired-end mismatch Mockrobiota
Paired-end contamination Mockrobiota
Paired-end unmatch Mockrobiota
D
Paired-end Correct Mockrobiota
0.0
0.2
0.4
0.6
0.8
1.0
Paired-end Over-merging Mockrobiota
Paired-end Over-Splitting Mockrobiota
E
Paired-end HC227_V3V4 ASV-ref Reference hit
Total Reference Number
0
50
100
150
200
Class
Correct
Overmerged
Oversplit
Missed
