# Supplementary material for: The unresolved struggle of 16S rRNA amplicon sequencing: a benchmarking analysis of clustering and denoising methods
Source: Environ Microbiome. 2025 May 13;20:51. doi: 10.1186/s40793-025-00705-6 (PMC12076876; doi:10.1186/s40793-025-00705-6)
Supplement: Supplementary file 2 — Supplementary Material 2: fig. 2: rarefaction curves and alluvial plots for both single-end and paired-end data. Rarefaction curves were illustrated for representing sequencing depth for denoising tools against ASV-ref and clustering methods against OTU-ref for both single-end and paired-end conditions respectively. Alluvial plots were utilized for visualizing the bacterial content across denoising and clustering algorithms for paired-end method. [file 40793_2025_705_MOESM2_ESM.pdf]

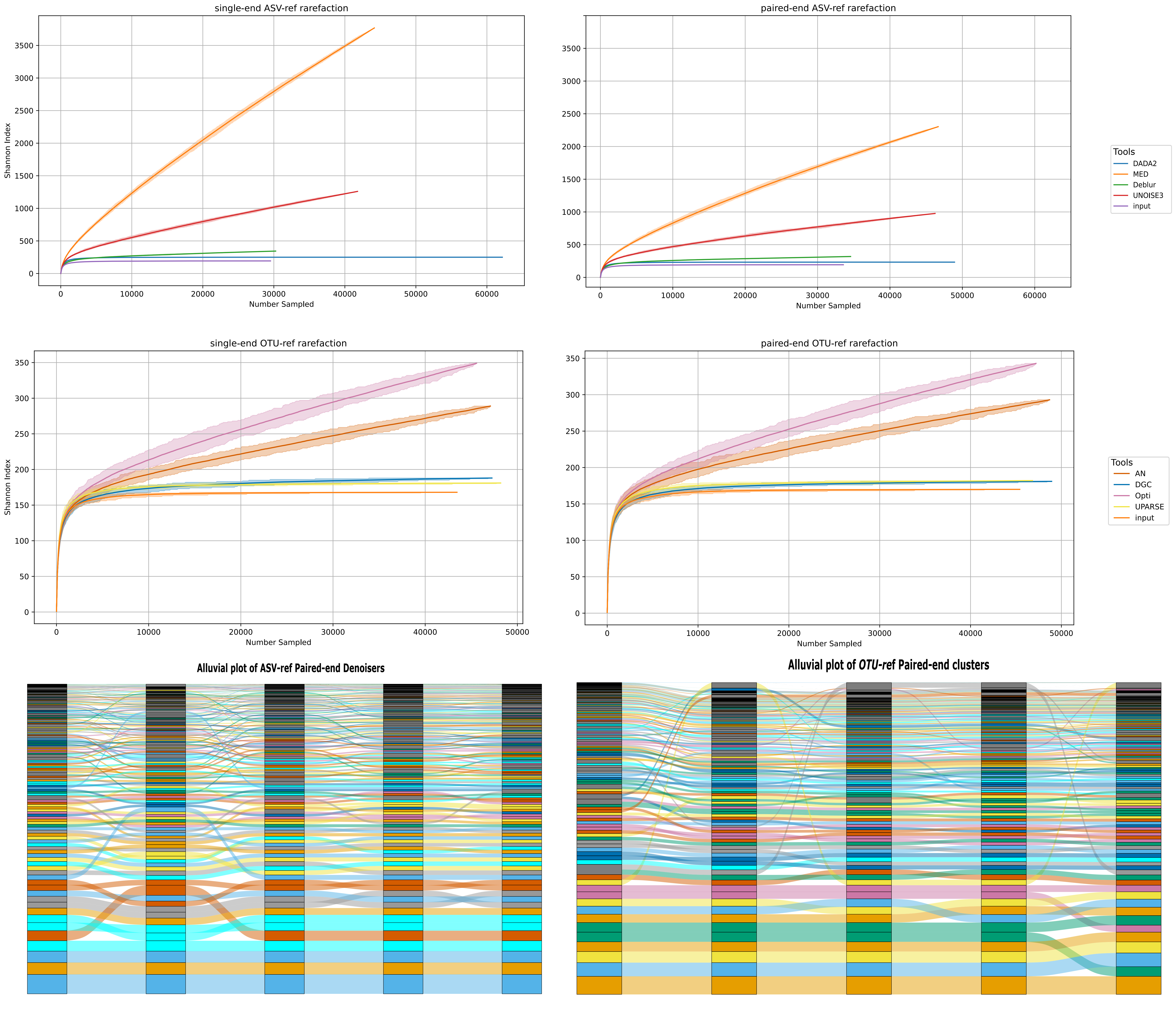
single-end ASV-ref rarefaction
paired-end ASV-ref rarefaction
Shannon Index
Number Sampled
Tools
DADA2
MED
Deblur
UNOISE3
input
single-end OTU-ref rarefaction
paired-end OTU-ref rarefaction
Shannon Index
Number Sampled
Tools
AN
DGC
Opti
UPARSE
input
Alluvial plot of ASV-ref Paired-end Denoisers
Alluvial plot of OTU-ref Paired-end clusters
