# Supplementary material for: The unresolved struggle of 16S rRNA amplicon sequencing: a benchmarking analysis of clustering and denoising methods
Source: Environ Microbiome. 2025 May 13;20:51. doi: 10.1186/s40793-025-00705-6 (PMC12076876; doi:10.1186/s40793-025-00705-6)
Supplement: Supplementary file 3 — Supplementary Material 3: fig. 3: illustration of HC227_V3V4 utilizing euclidian distance and alluvial plots for visualizing bacterial content for denoising and clustering methods. (A) Beta Diversity for denoising algorithms against ASV-ref data for both single-end and paired-end methods. (B) Beta Diversity for clustering algorithms against OTU-ref for both single-end and paired-end methods. C & D) stacked bar plots illustrating read count for all algorithms for Mockrobiota and HC227_V3V4 mock communities considering single-end and paired-end conditions. E) Alluvial plots were utilized for visualizing the bacterial content across denoising and clustering algorithms for single-end method. [file 40793_2025_705_MOESM3_ESM.pdf]

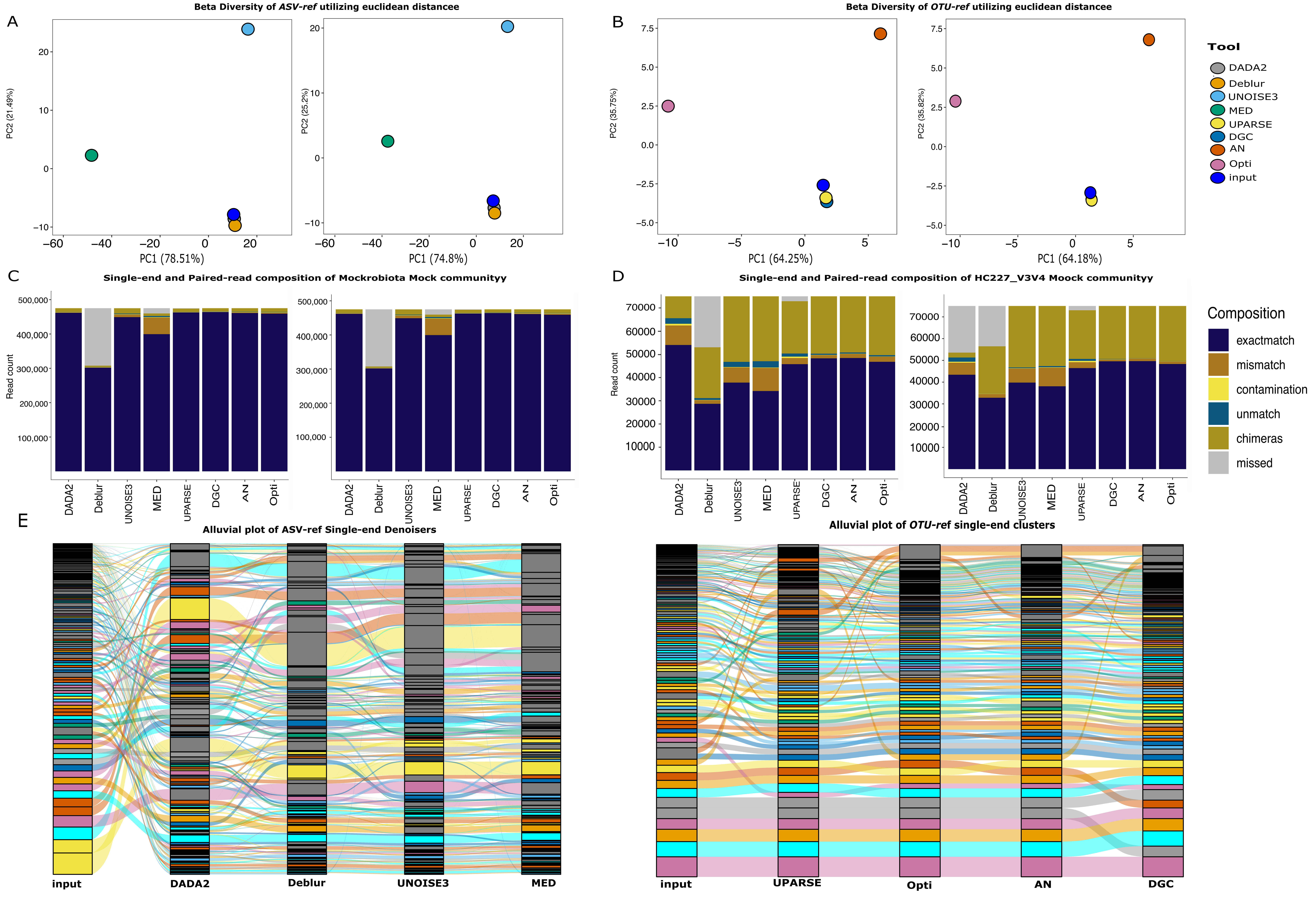

A
Beta Diversity of ASV-ref utilizing euclidean distancee
PC2 (21.49%)
PC1 (78.51%)
PC2 (25.2%)
PC1 (74.8%)
B
Beta Diversity of OTU-ref utilizing euclidean distancee
PC2 (35.75%)
PC1 (64.25%)
PC2 (35.82%)
PC1 (64.18%)
Tool
DADA2
Deblur
UNOISE3
MED
UPARSE
DGC
AN
Opti
input
C
Single-end and Paired-read composition of Mockrobiota Mock communityy
Read count
DADA2
Deblur
UNOISE3
MED
UPARSE
DGC
AN
Opti
D
Single-end and Paired-read composition of HC227_V3V4 Moock communityy
Read count
Composition
exactmatch
mismatch
contamination
unmatch
chimeras
missed
E
Alluvial plot of ASV-ref Single-end Denoisers
input
DADA2
Deblur
UNOISE3
MED
Alluvial plot of OTU-ref single-end clusters
input
UPARSE
Opti
AN
DGC
